# The Imperative of Data Transparency for Pursuing Scientific Truth about COVID-19 Vaccination and Excess Deaths

**DOI:** 10.31662/jmaj.2025-0522

**Published:** 2026-04-24

**Authors:** Hideki Kakeya, Takeshi Nitta, Yukari Kamijima, Takayuki Miyazawa

**Affiliations:** 1 Institute of Systems and Information Engineering, University of Tsukuba, Tsukuba, Ibaraki, Japan; 2 Research Institute for Biomedical Sciences, Tokyo University of Science, Yamazaki, Noda, Chiba, Japan; 3 Faculty of Pharmaceutical Sciences, Tokyo University of Science, Niijuku, Katsushika-ku, Tokyo, Japan; 4 Kyoto Animal Human Organism Research Institute, Shimomaruyacho, Nakagyo, Kyoto, Japan

**Keywords:** COVID-19 vaccination, excess mortality, data transparency, IgG4 immune response, public health policy, epidemiological data

## To the Editor,

We appreciate the opportunity to respond to the letter by Kutsuna and Suzuki ^(1)^, which comments on our opinion paper ^(2)^. Their letter raises important points for scientific discussion. However, it appears to misunderstand the main intent of our article, which was to call for the Ministry of Health, Labour and Welfare (MHLW) to resume publication of coronavirus disease 2019 (COVID-19) vaccination and mortality data that had previously been available. We presented several hypotheses as possible explanations for the observed increase in excess deaths. These hypotheses should not be prematurely dismissed when scientifically examining the alarming situation of increased deaths among the public. This is because the long-term safety of COVID-19 vaccines has been a matter of concern since the start of vaccination campaigns and remains one of the unresolved issues ^(3)^.

COVID-19 vaccines have triggered immune responses not initially anticipated. One such example is the high production of immunoglobulin G4 (IgG4). Beyond reference 11 in our paper ^(4)^, other reports have demonstrated that multiple doses of mRNA vaccines significantly induce IgG4 production ^(5), (6), (7), (8)^. Many researchers have expressed interest and concern about this unexpected immune response ^(9)^. Indeed, many studies throughout the COVID-19 pandemic have reported an association between high IgG4 production and an increased risk of severe acute respiratory syndrome coronavirus 2 (SARS-CoV-2) infection ^(10), (11), (12)^. The potential impact of high IgG4 production following vaccination on SARS-CoV-2 infection and other diseases should not be dismissed as an overstatement; rather, it warrants serious consideration and discussion by many researchers in both clinical and basic fields.

We concur with Kutsuna and Suzuki’s view that “most solid tumors progress over a period of several years to decades before leading to mortality.” However, one of the cancers we discussed in our opinion paper―pancreatic cancer―has been reported to show poorer treatment outcomes among repeatedly vaccinated populations ^(7)^. A recent peer-reviewed paper from Korea also reports an association between COVID-19 vaccination and cancers ^(13)^. Therefore, we cannot rule out the possibility that vaccination may contribute to excess mortality by exacerbating preexisting cancers.

Furthermore, Kutsuna and Suzuki cite aging and several transient factors as possible causes of excess mortality. However, in Japan, average life expectancy declined significantly in 2022 and has continued to stagnate since. Naturally, a decline in average life expectancy cannot be attributed to population aging. Identifying the factors that contribute to the stagnation of Japan’s life expectancy―without any preconceived notions about whether vaccines are involved or not―is a task that Japan’s academic community should vigorously pursue.

Kutsuna and Suzuki claim that our article “misuses data from the Health Center Real-time Information-sharing System on COVID-19 (HER-SYS),” arguing that cases without recorded vaccination dates were automatically counted as “unvaccinated” and that such data must not be used to evaluate vaccine effectiveness. Our article, however, did not use HER-SYS data to estimate vaccine effectiveness or safety, nor did it assume causality; rather, it highlighted structural limitations in data management by the MHLW and the difficulty of external validation after data provision was suspended. While we share their concern regarding the incompleteness of HER-SYS vaccination records, some epidemiologists in Japan have employed HER-SYS data for logistic modeling of vaccination status ^(14)^. The criticism should therefore be directed at such studies rather than at our article.

Kutsuna and Suzuki also assert that “without individual-level data linking vaccination status to outcomes, causal inference cannot be made.” That is correct; however, the Kutsuna-Suzuki letter proceeds to infer that the excess deaths must have causes other than vaccination, which is a causal inference in itself. Furthermore, the claim that our paper draws conclusions without adequate evidence is inaccurate. Our paper explicitly outlines several hypotheses, accompanied by the clear caveat that “the truth has yet to be established.” We believe that scholars genuinely dedicated to scientific inquiry should strive for precision and balance in their critiques.

Several preliminary analyses have explored possible associations between vaccination and excess mortality ^(15), (16), (17)^. These exploratory findings underscore the importance of transparent data access to enable independent verification. If vaccination were completely unrelated to excess mortality, the release of more detailed information that could refute such an association would help those who promote vaccination. It is therefore our academic and ethical responsibility to call for conditions that allow for proper causal investigation ― including access to data and independent evaluation. We believe that all scientists would agree on the importance of pursuing public health policy based on evidence-based medicine. Studies reviewing and reflecting on public health policies during the COVID-19 pandemic are underway worldwide ^(18)^, and similar efforts are urgently needed in Japan.

The failure caused by reluctance to disclose information has recently occurred in the field of epidemiology in Japan. The peer-reviewed paper by Kayano et al., ^(14)^ published in Scientific Reports, claimed, based on counterfactual simulation, that Japan’s 2021 vaccination program saved 350,000 lives. This figure was questioned, and repeated requests were made for the release of the simulation’s source code. More than a year and a half after publication, the corresponding author eventually complied, issued a correction, and released the source code. However, it was then found that entirely different computational methods had been used for the scenarios with and without a vaccination program. When the same method used for the “no vaccination” scenario was applied to simulate the real-world vaccination program, the model produced three times as many infections as actually occurred ^(19)^.

As this example shows, data transparency is of paramount importance in science. We respectfully urge Japan’s academic and medical communities to foster a culture of transparency. Only through open data sharing can balanced scientific discussion on vaccination and public health be achieved.

## Article Information

### Acknowledgments

The authors thank Mr. William Brooks for comments and suggestions on the manuscript.

### Author Contributions

Drafted the original manuscript: Hideki Kakeya. Revised it: Takeshi Nitta and Yukari Kamijima. Supervised the work from a virological perspective: Takayuki Miyazawa.

### Conflicts of Interest

None
